# Are public health professionals prepared for public health genomics? A cross-sectional survey in Italy

**DOI:** 10.1186/1472-6963-14-239

**Published:** 2014-05-28

**Authors:** Carolina Marzuillo, Corrado De Vito, Maddalena D’Addario, Paola Santini, Elvira D’Andrea, Antonio Boccia, Paolo Villari

**Affiliations:** 1 Department of Public Health and Infectious Diseases, Sapienza University of Rome, Piazzale Aldo Moro 5, Rome 00185, Italy

**Keywords:** Public health genomics, Predictive genetic testing, Public health professionals, Cross-sectional survey, Knowledge and attitudes, Training needs

## Abstract

**Background:**

Public health genomics is an emerging multidisciplinary approach, which aims to integrate genome-based knowledge in a responsible and effective way into public health. Despite several surveys performed to evaluate knowledge, attitudes and professional behaviors of physicians towards predictive genetic testing, similar surveys have not been carried out for public health practitioners. This study is the first to assess knowledge, attitudes and training needs of public health professionals in the field of predictive genetic testing for chronic diseases.

**Methods:**

A self-administered questionnaire was used to carry out a cross-sectional survey of a random sample of Italian public health professionals.

**Results:**

A response rate of 67.4% (797 questionnaires) was achieved. Italian public health professionals have the necessary attitudinal background to contribute to the proper use of predictive genetic testing for chronic diseases, but they need additional training to increase their methodological knowledge. Knowledge significantly increases with exposure to predictive genetic testing during postgraduate training (odds ratio (OR) = 1.74, 95% confidence interval (CI) = 1.05–2.88), time dedicated to continuing medical education (OR = 1.53, 95% CI = 1.14–2.04) and level of English language knowledge (OR = 1.36, 95% CI = 1.07–1.72). Adequate knowledge is the strongest predictor of positive attitudes from a public health perspective (OR = 3.98, 95% CI = 2.44–6.50). Physicians show a lower level of knowledge and more public health attitudes than other public health professionals do. About 80% of public health professionals considered their knowledge inadequate and 86.0% believed that it should be improved through specific postgraduate training courses.

**Conclusions:**

Specific and targeted training initiatives are needed to develop a skilled public health workforce competent in identifying genomic technology that is ready for use in population health and in modeling public health genomic programs and primary care services that need to be developed, implemented and evaluated.

## Background

The decade following the completion of the human genome project has been marked by divergent claims about the utility of genomics for public health purposes. Some public health advocates contend that interventions based on environmental changes will be more effective than those focused on individual behavior change. By contrast, those supportive of a role for public health genomics argue that increasing knowledge of genomics and molecular pathology could unlock effective diagnostic techniques and treatments, as well as better target public health interventions [1-6].

Public health practitioners from academic, government and other organizations have taken a proactive leadership role in assessing the relevance of DNA technology to population health and to community health interventions [7-10]. Among the priorities of the public health genomics movement, there is the assurance of an adequate public health capacity on genomics [11-13]. Appropriate capacity building and the development of a skilled public health workforce competent in the differentiation between genomic technology that is ready for use in population health and technology that is not ready for prime time is a fundamental issue to ensure the appropriate use of genomic information for health promotion and prevention of diseases. Predictive genetics have currently few applications in clinical practice, but the scenario is likely to change in the future. In Italy, as well as in other countries, predictive tests have raised interest in public health, particularly in the case of high-penetrance genetic variants associated with common types of cancer (breast/ovarian and colorectal cancer) and familiar hypercholesterolemia. These predictive genetic tests, if used appropriately, have been demonstrated to be efficacious and cost-effective [14].

Many surveys have been conducted to assess knowledge, attitudes and professional behavior of physicians toward predictive genetic testing for chronic diseases [15-37]. This study is the first to be carried out on public health practitioners. It was conducted in Italy, where a specific public health genomics policy is being developed [38]. Predictive medicine is one of the four macro-areas of intervention of the 2010–2012 National Preventive Plan, which foresees the drawing up of a dedicated National Plan for Public Health Genomics (PHG-NP). This national plan, which defines the actions to be taken at central level in order to implement a stewardship governance model to best translate genomics in clinical practice, has been recently approved [39].

## Methods

### Participants

A link to a self-administered anonymous online questionnaire was e-mailed in 2010 to 1,200 public health professionals randomly selected from the register of the Italian Society of Hygiene, Preventive Medicine and Public Health (S.It.I.). An accompanying cover letter outlined the details of the study and assured the participants of anonymity. The online questionnaire could only be submitted once per person. Up to two reminder e-mails containing the link to the online questionnaire were sent to non-responders 3 and 6 months after the initial e-mail. To maximize the response rate, telephone calls were made to all subjects before each of the follow-up mailings. A total of 60 public health professionals could not be contacted by telephone because their numbers were not available.

The design of the study allowed the research team to identify non-responders and to compare their demographic and professional characteristics with those of the responders. After the comparisons were made, the identification codes were destroyed, thereby maintaining total anonymity for all participants.

The study was in compliance with the Declaration of Helsinki and ethical approval was obtained by the Ethical Committee of Azienda Policlinico Umberto I (Rome, Italy).

### Survey instrument

The questionnaire (see the complete questionnaire in the Additional file 1 for more details) comprised a series of questions designed to assess socio-demographic and professional characteristics, knowledge and public health attitudes towards predictive genetic tests for chronic diseases, and self-estimated level of knowledge and training needs.

Knowledge about predictive genetic tests for chronic diseases was investigated through seven questions using a three-point Likert scale (“agree”, “uncertain”, and “disagree”). The same Likert three-point scale was used to assess the participants’ public health attitudes, i.e. attitudes that may predispose individuals to adopt or reject specific public health-related behaviors [40]. Public health professionals were finally asked to assess their own level of knowledge on a four-point scale (“inadequate”, “sufficient”, “good”, and “excellent”) and answer four questions (each with “yes/no” answers) on training needs.

Extensive pre-administration piloting was conducted with a convenience sample of 40 physicians similar to the study population to ensure practicability, validity and interpretation of answers. On the basis of the comments and suggestions obtained from the pilot study, the questionnaire was revised before distribution to the study sample. Instrument revision included changes to questionnaire item wording and format. Items were only included in the survey instrument if there was consensus on their meaning.

### Statistical analysis

Multiple logistic regression was performed to identify predictors of knowledge (Model 1) and positive public health attitudes (Model 2). For the purpose of analysis, the outcome variables “knowledge” and “attitudes” originally consisting of multiple categories were collapsed into two levels. In brief, for the knowledge variable, responders were divided into those who agreed with all correct responses versus all others, while for attitudes, public health practitioners were grouped into those who showed a positive public health attitude in all questions versus all others. The following predictor variables were initially tested in both models: geographical area of professional activity; gender; age; medical degree; professional activity; exposure to predictive genetic testing during undergraduate/postgraduate courses; knowledge of the English language; internet access in the workplace; hours per week dedicated to continuing medical education; and reception in the previous year of specific informative from institutional sources. In the model concerning attitudes, the variable “adequate knowledge of genetic testing”—dichotomized as above—was also included.

Multiple logistic regression models were built using the strategy suggested by Hosmer and Lemeshow [41]. Each variable was examined by univariate analysis using the appropriate statistical test (Student’s t-test or χ2 test), and was included in the model when the *P*-value was less than 0.25. Subsequently, multivariate logistic regression with backward elimination of any variable that did not contribute to the model on the grounds of the Likelihood Ratio test (cut-off, *P* = 0.05) was performed. Variables whose exclusion altered the coefficient of the remaining variables were kept in the model. Interaction terms were tested using a cut-off significance level of 0.15. Adjusted odds ratios (ORs) and 95% confidence intervals (CIs) were calculated. All statistical calculations were performed using Stata version 8.0 (Stata Corporation, College Station, TX, USA).

## Results

Out of the original sample of 1,200 public health professionals 797 answered giving an overall response rate of 67.4% (see Table 1 for demographic and professional characteristics of the responding public health professionals). Responders and non-responders were comparable in terms of demographic and professional characteristics (gender, age, type of degree and professional activity; *P* >0.05). Most responders were female (53.9% vs. 52.6% of non-responders), with a mean age of 47.5 (±10.9) years (48.7 years for non-responders), had a medical degree (79.7% vs. 75.9%) and were employed in Public Health Services of the Italian National Health Service (64.1% vs. 69.0%).

**Table 1:** Demographic and professional characteristics of the responding public health professionals

**Variables**	**N.**	**%**
*Geographical area* (785)^a^		
North	244	31.1
Center	184	23.4
South	225	28.7
Islands	132	16.8
*Gender* (790)^a^		
Female	426	53.9
Male	364	46.1
*Age, years* (790)^a^		
≤30	52	6.6
31-40	191	24.2
41-50	182	23.0
51-60	297	37.6
≥61	68	8.6
*Type of degree* (786)^a^		
Medicine	626	79.7
Biology	83	10.6
Nursing	27	3.4
Other health professions degrees	25	3.1
Other degrees	25	3.2
*Exposure to predictive genetic testing during undergraduate training* (797)^a^		
No	635	79.7
Yes	162	20.3
*Exposure to predictive genetic testing during postgraduate training* (797)^a^		
No	603	75.7
Yes	194	24.3
*English language knowledge* (789)^a^		
Very low	126	16.0
Low	200	25.3
Intermediate	270	34.2
Good	153	19.4
Excellent	40	5.1
*Internet available in the workplace* (797)^a^		
No	44	5.5
Yes	753	94.5
*Hours per week dedicated to continuing medical education* (797)^a^		
<1	114	14.3
1-5	440	55.2
6-10	166	20.8
>10	77	9.7
*Reception in the previous year of specific information concerning predictive genetic testing from institutional sources* (797)^a^		
No	583	73.1
Yes	214	26.9

^a^Number of public health professionals responding to the question.

No statistically significant differences were detected between our sample and the study population, since 52.8% of the S.It.I. members are female and have a similar age distribution (S.It.I., personal communication, 2012). The proportions of S.It.I. members with a medical degree and employed in Public Health Services of the Italian NHS were 77.1% and 66.4%, respectively.

A minority of responders were exposed to predictive genetic testing during undergraduate (20.3%) or postgraduate (24.3%) training. Knowledge of the English language appeared to be relatively poor, as 41.4% of subjects indicated “very low” or “low” levels of English. The majority of the sample (55.2%) dedicated 1–5 hours per week to continuing medical education. Less than one third of the responders (26.9%) received specific informative about predictive genetic testing from institutional sources in the previous year (Table 1).

Knowledge of predictive genetic testing for chronic diseases appeared adequate among public health professionals in Italy (Table 2). Almost all responders recognized that predictive genetic tests could identify individuals at higher risk of developing diseases, and the majority correctly agreed with definitions of analytic validity (70.6%), clinical validity (63.0%) and clinical utility (68.0%) of predictive genetic tests. Three-quarters of the sample acknowledged the importance of genetic counseling, but only half were aware of the availability of evidence-based recommendations/guidelines for some predictive genetic tests (Table 2). Only 10.2% of the responders answered all seven questions about predictive genetic testing correctly and this knowledge was significantly associated with exposure to predictive genetic testing during postgraduate training (OR = 1.74, 95% CI = 1.05–2.88), with time dedicated to continuing medical education (OR = 1.53, 95% CI = 1.14–2.04) and with level of English language knowledge (OR = 1.36, 95% CI = 1.07–1.72). Being a physician was a negative predictor of adequate knowledge (OR = 0.54, 95% CI = 0.32–0.92) (Model 1 in Table 3).

**Table 2:** Knowledge of the responding public health professionals regarding predictive genetic testing for chronic diseases

**Variables**	**Agree**	**Uncertain**	**Disagree**
	**%**	**%**	**%**
*Predictive genetic tests are able to identify genotypes which themselves do not cause the disease but modify the risk of developing it* (788)^a^	**86.6**	9.0	4.4
*Lifestyles, socioeconomic factors and pollution exposure cannot modify or influence the risk of disease due to a genetic predisposition* (788)^a^	14.2	10.4	**75.4**
*The analytic validity of a predictive genetic test is related to the accuracy of the laboratory test in identifying a specific genetic characteristic* (788)^a^	**70.6**	25.6	3.8
*The clinical validity of a predictive genetic test is related to the power of the test to quantify the risk of developing the disease* (787)^a^	**63.0**	24.1	12.8
*The clinical utility of a predictive genetic test is related to the power of the test to improve the health status of the subject* (787)^a^	**68.0**	20.8	11.2
*Performing predictive genetic tests should not necessarily be associated with genetic counseling that includes information, informed consent, and discussion of the results* (787)^a^	11.8	13.2	**75.0**
*Recommendations/guidelines produced by national/international organizations about the use of some predictive genetic tests already exist* (786)^a^	**47.1**	49.1	3.8

Note: Percentages referring to correct answers are in bold.

^a^Number of public health professionals responding to the question.

**Table 3:** Determinants of public health professionals’ knowledge and attitudes concerning predictive genetic testing for chronic diseases

**Variables**	**OR**	**95% CI**
Model 1: Knowledge about predictive genetic testing^a^.		
Hours per week dedicated to continuing medical education (<1 = 0; 1-5 = 1; 6-10 = 2; > 10 = 3)^b^	1.53	1.14 – 2.04
Exposure to predictive genetic tests during postgraduate training (No = 0; Yes = 1)	1.74	1.05 – 2.88
English language knowledge (Very low = 0; low = 1; intermediate = 2; good = 3; excellent = 4)^b^	1.36	1.07 – 1.72
Medical degree (No = 0; Yes = 1)	0.54	0.32 – 0.92
Model 2: Attitudes about predictive genetic testing^c^.		
Hours per week dedicated to continuing medical education (<1 = 0; 1-5 = 1; 6-10 = 2; > 10 = 3)^b^	1.31	1.06 – 1.61
Exposure to cancer genetic tests during undergraduate training (No = 0; Yes = 1)	1.53	1.03 – 2.26
Medical degree (No = 0; Yes = 1)	2.17	1.34 – 3.51
Knowledge about predictive genetic testing (Not adequate = 0; adequate = 1)^a^	3.98	2.44 – 6.50

Note: OR: Odds Ratio. CI: Confidence Interval.

^a^Physicians were classified as those who answered correctly all questions on predictive genetic testing (Table 2) vs. all others.

^b^Variable modeled as ordinal because linearity was assessed.

^c^Public health professionals were divided into those who showed positive public health attitudes in all questions (Table 4) vs. all others.

Most of responders showed positive attitudes—from a public health perspective—towards predictive genetic testing for chronic diseases (Table 4). The majority of the responders disagreed that predictive genetic tests should be introduced into clinical and public health practice even without health interventions with proven efficacy (57.9%) and 55.7% of them agreed that predictive genetic testing should be performed only if there is evidence of cost-effectiveness. The vast majority of public health professionals recognized that predictive genetic testing should be included in wider prevention strategies taking into account other available health interventions (90.5%) and acknowledged the importance of ethical, legal and social implications (82.5%) (Table 4). A total of 24.3% of public health professionals showed a positive public health attitude in all six questions, and this dichotomization was used to identify as significant predictors (i) adequate knowledge (OR = 3.98, 95% CI = 2.44–6.50), (ii) exposure to predictive genetic testing during underunder training (OR = 1.53, 95% CI = 1.03–2.26) and (iii) time dedicated to continuing medical education (OR = 1.31, 95% CI = 1.06–1.61). Physicians were more likely to show positive public health attitudes than other public health professionals were (OR = 2.17, 95% CI = 1.34–3.51) (Model 2 in Table 3).

**Table 4:** Public health attitudes of the responding public health professionals towards predictive genetic testing for chronic diseases

**Variables**	**Agree**	**Uncertain**	**Disagree**
	**%**	**%**	**%**
*Predictive genetic tests increase prevention opportunities for chronic diseases* (788)^a^	**76.5**	17.5	6.0
*Predictive genetic tests able to identify an increased risk of developing a disease should be introduced in the clinical and public health practice even without health interventions with proven efficacy* (788)^a^	22.0	20.2	**57.9**
*Predictive genetic tests should be introduced in the clinical and public health practice only if economic evaluations show cost-effectiveness ratios favorable compared with alternative health interventions* (787)^a^	**55.7**	26.0	18.3
*Authoritative and evidence based guidelines are needed for the appropriate use of predictive genetic tests* (788)^a^	**95.2**	4.2	0.6
*Predictive genetic tests can contribute efficaciously to health promotion and disease prevention only if included in wider strategies taking into account the other available health interventions* (788)^a^	**90.5**	6.9	2.7
*The implementation of predictive genetic testing in the clinical and public health practice, being a medical matter, should not take into account ethical, legal and social implications* (788)^a^	8.1	9.4	**82.5**

Note: Percentages referring to answers denoting a positive public health attitude are in bold.

^a^Number of public health professionals responding to the question.

About 80% of public health professionals considered their knowledge on predictive genetic testing for chronic diseases to be inadequate. The majority of responders agreed there is a need for increased training on predictive genetic testing for chronic diseases during undergraduate (90.8%) or postgraduate courses (94.6%). Almost all responders (94.6%) believed that their knowledge should be improved, and 86.0% believed that specific post-training courses in predictive genetic testing for chronic diseases should be implemented (data not shown).

## Discussion

A specific health policy concerning public health genomics is currently being developed in Italy by the Ministry of Health [38]. A dedicated National Plan for Public Health Genomics (PHG-NP) has recently been drawn-up that addresses in depth how to translate genomics knowledge into public health [39]. To achieve the strategic objectives of the PHG-NP, systematic health technology assessment of predictive genetic test for complex diseases, promotion of genomic education among physicians and the general public and the development of capacity building among all potential stakeholders for the health care appropriate provision and management of predictive genetic testing will be needed. The public health community is therefore called at playing a proactive role to integrate genome-based knowledge into public health in a responsible and effective way, also anticipating in a certain way the increase in the health service requirements that is likely to occur in the future [14].

To our knowledge, this survey is the first to be conducted on public health practitioners. The results show that the Italian public health community has the necessary attitudinal background to contribute to the proper use of predictive genetic testing for chronic diseases, but that an additional training to increase methodological knowledge is needed. Despite more positive public health attitudes, public health physicians have more gaps in their knowledge than other public health professionals (who are mainly biologists), reflecting possible deficiencies in the genetics components of current medical curricula in Italy. Compared with Italian physicians, who previously showed significant training needs in the field of efficacy, effectiveness and economic evaluation of health interventions [42,43], public health attitudes towards predictive genetic testing appear to be more positive among public health practitioners. For example, the percentage of public health professionals who agree that the selection of predictive genetic testing to be delivered to the population should be based on the principles of efficacy and cost-effectiveness are higher than those found among Italian physicians in another survey [44]. Globally, the public health community in Italy appears to be more prepared than physicians for a responsible and appropriate introduction of DNA-technology into health care and public health practice.

Previous surveys carried out in the United States among health educators showed that education and training influence public health genomics knowledge and attitudes [45,46]. The results of the present survey are consistent with these findings. Exposure to predictive genetic testing during undergraduate and postgraduate training and time dedicated to continuing medical education are significant determinants of both knowledge and positive attitudes. Adequate knowledge is the strongest predictor of positive public health attitudes and there is a high level of interest in further education and training to improve knowledge and skills in this field. Overall, the results of this survey clearly indicate that there is a strong need for specific and targeted training initiatives for the public health workforce.

However, lessons drawn from many areas of medicine indicate that education alone does not necessarily translate into effective and appropriate adoption of innovative practice [47,48]. Organizational changes are needed within the health care system to provide these services effectively and efficiently. In theory, predictive genetic testing can be used in population screening programs led by public health professionals or for early case detection and intervention in primary care settings. Today, there is a limited evidence base to support either genetic population screening programs or a personalized individual predictive genetic testing, but this scenario is likely to change to a large extent in the future [14,49-60]. The small number of clinical geneticists in practice will limit their ability to participate in the care associated with an expending menu of genetic tests [51]. Public health professionals could and should play an important role in the “honest broker” evaluation process that can discriminate those genomic applications that can improve health from those that are likely to result in potential harm and unnecessary health care expenditure through premature use. Most importantly, public health professionals can contribute to the modeling of public health genomic programs and primary care services that need to be developed, implemented, and evaluated [61-63].

The main limitation of this survey concerns the generalizability of its results. While the sample surveyed was representative of the study population and the response rate was high, differences between the Italian and other European and non-European public health workforces are likely to exist. Despite the fact that public health is one of the established specialties in the European Union (EU) [64] and, consequently, a specialist trained in one EU country will be recognized as a specialist in all EU countries, non-uniformity of public health curricula is a recognized problem [65]. Moreover, public health today requires a multidisciplinary workforce [66], and the non-medical component of the public health workforce – in Italy represented mainly by biologists and people with health profession degrees – could vary among countries. Therefore, the knowledge, public health attitudes and training needs in the field of public health genomics should also be assessed in other countries, hopefully within specific national health policy frameworks.

## Conclusions

In conclusion, the results of this survey show that the Italian public health community has the necessary attitudinal background to contribute to the proper introduction and use of predictive genetic testing for chronic diseases, but some knowledge gaps exist that should be filled through appropriate training. A specific policy of public health genomics is currently being developed at a national level by the Ministry of Health in Italy [38], with three major mainstays: (i) systematic health technology assessments of genetic tests for complex diseases; (ii) promotion of genomic education in physicians and capacity building; and (iii) promotion of basic genomic health literacy for the general population. The implementation of such a policy is an obligation for public health professionals. They will lose credibility if, on the one hand, they promote health literacy enabling and empowering individuals for decision making, while, on the other hand, they ignore genomic and genetic knowledge, thus missing opportunities to provide evidence-based public health interventions.

## Abbreviations

S.It.I.: Italian Society of Hygiene, Preventive Medicine and Public Health; OR: Odds ratio; 95% CI: 95% confidence interval; PHG-NP: National Plan for Public Health Genomics; EU: European Union.

## Competing interests

The authors declare that they have no competing interests.

## Authors’ contributions

CM made substantial a contribution to the study design and acquisition of data, and helped to analyze data and draft the manuscript. CDV participated in the design of the study, performed the statistical analysis and helped to draft the manuscript. EDA, MDA and PS participated in the design of the study and in the acquisition of data. AB participated in the design of the study, and was involved in drafting the manuscript and revising it critically for important intellectual contents. PV conceived the study, participated in its design and coordination and drafted the manuscript. All authors read and approved the final version of the manuscript.

## Pre-publication history

The pre-publication history for this paper can be accessed here:

http://www.biomedcentral.com/1472-6963/14/239/prepub

## Supplementary Material

Additional file 1 Questionnaire on knowledge, attitudes and training needs of public health professionals on the use of predictive genetic tests. Click here for file
