# Supplementary material for: Are public health professionals prepared for public health genomics? A cross-sectional survey in Italy
Source: BMC Health Serv Res. 2014 May 28;14:239. doi: 10.1186/1472-6963-14-239 (PMC4064825; doi:10.1186/1472-6963-14-239)
Supplement: Additional file 1 — Questionnaire on knowledge, attitudes and training needs of public health professionals on the use of predictive genetic tests. [file 1472-6963-14-239-S1.doc]

**SURVEY ON KNOWLEDGE, ATTITUDES AND TRAINING NEEDS OF PUBLIC HEALTH PROFESSIONALS ON THE USE OF PREDICTIVE GENETIC TESTS**

***Make an X or respond briefly when instructed;***

***tick one box only, unless otherwise specified.***

**A. PERSONAL DETAILS**

# *a1*. Birth year ________________ gender M ❒ F ❒

**B. PROFESSIONAL ACTIVITY**

*b1.* YEAR OF DEGREE _______________________________________________

*b2.* TYPE OF DEGREE__________________________________________

*B3*. POSTGRADUATE TRAINING *(MULTIPLE ANSWERS ARE ALLOWED)*:

a) Postgraduate School ❒ Type:____________________________________________________

b) Ph. D. ❒ Type:____________________________________________________

c) Master ❒ Type: ____________________________________________________

d) Advanced postgraduate ❒ Type: ____________________________________________________

training courses

e) Other ❒ Please, specify: ____________________________________________

*B4*. CURRENT SETTING OF PROFESSIONAL ACTIVITY

Please, specify _______________________________________________________________________________

___________________________________________________________________________________________

___________________________________________________________________________________________

___________________________________________________________________________________________

*b5.* HOW MANY YEARS do you HELD YOUR CURRENT professional activity?__________________

*b6.* in which CITY DO you conduct your professional activity?___________________________

# *B7*. HAVE PREDICTIVE GENETIC TESTING BEEN TREATED DURING YOUR UNDERGRADUATE TRAINING?

# NO ❒ YES ❒

# *B8*. HAVE PREDICTIVE GENETIC TESTING BEEN TREATED DURING YOUR POSTGRADUATE TRAINING?

NO ❒ YES ❒

**C. ACCESS TO CONTINUING MEDICAL EDUCATION**

*C1*. HOW WOULD YOU RATE YOUR LEVEL OF ENGLISH LANGUAGE KNOWLEDGE?

very low ❒ low ❒ intermediate ❒ good ❒ excellent ❒

*C2.* IS THERE AN INTERNET ACCESS AVAILABLE IN YOUR WORKPLACE?

NO ❒ YES ❒

*C3*. IS THERE A SCIENTIFIC LIBRARY AVAILABLE IN YOUR WORKPLACE?

NO ❒ YES ❒

*C4*. HOW MANY HOURS PER WEEK DO YOU SPEND IN CONTINUING MEDICAL EDUCATION?

<1 hour per week ❒ 1-5 hours per week ❒ 6-10 hours per week ❒ >10 hours per week ❒

*C5*. DURING THE LAST YEAR DID YOU RECEIVED INFORMATION MATERIAL ON PREDICTIVE GENETIC TESTING BY THE FOLLOWING AUTHORITIES?

MINISTRY OF HEALTH NO ❒ YES❒

REGION NO ❒ YES❒

LOCAL HEALTH AUTHORITY NO ❒ YES❒

BOARD OF PHYSICIANS NO❒ YES❒

SCIENTIFIC ASSOCIATIONS NO❒ YES❒

OTHER NO❒ YES❒ (Please, specify) ___________________

*C6*. DURING THE LAST YEAR DID YOU RECEIVED ADVERTISING MATERIAL ON PREDICTIVE GENETIC TESTING?

NO ❒ YES ❒

IF YES, FROM WHICH SOURCES?: _________________________________

***D. KNOWLEDGE***

| **Agree Uncertain Disagree** | |
| --- | --- |
| *D1*. Predictive genetic tests are able to identify genotypes which themselves do not cause the disease but modify the risk of developing it  *D2*. Lifestyles, socioeconomic factors and pollution exposure cannot modify or influence the risk of disease due to a genetic predisposition  *D3.* The analytic validity of a predictive genetic test is related to the accuracy of the laboratory test in identifying a specific genetic characteristic  *D4*. The clinical validity of a predictive genetic test is related to the power of the test to quantify the risk of developing the disease  *D5*. The clinical utility of a predictive genetic test is related to the power of the test to improve the health status of the subject  *D6.* Performing predictive genetic tests should not necessarily be associated with genetic counseling that includes information, informed consent, and discussion of the results  *D7.* Recommendations/guidelines produced by national/international organizations about the use of some predictive genetic tests already exist | ❒ ❒ ❒  ❒ ❒ ❒  ❒ ❒ ❒  ❒ ❒ ❒  ❒ ❒ ❒  ❒ ❒ ❒  ❒ ❒ ❒ |

**E. ATTITUDES**

| **Agree Uncertain Disagree** | |
| --- | --- |
| *E1*. Predictive genetic tests increase prevention opportunities for chronic diseases  *E2*. Predictive genetic tests able to identify an increased risk of developing a disease should be introduced in the clinical and public health practice even without health interventions with proven efficacy  *E3*. Predictive genetic tests should be introduced in the clinical and public health practice only if economic evaluations show cost-effectiveness ratios favorable compared with alternative health interventions  *E4*. Authoritative and evidence based guidelines are needed for the appropriate use of predictive genetic tests  E5. Predictive genetic tests can contribute efficaciously to health promotion and disease prevention only if included in wider strategies taking into account the other available health interventions  *E6.* The implementation of predictive genetic testing in the clinical and public health practice, being a medical matter, should not take into account ethical, legal and social implications | ❒ ❒ ❒  ❒ ❒ ❒  ❒ ❒ ❒  ❒ ❒ ❒  ❒ ❒ ❒  ❒ ❒ ❒ |

#### F. TRAINING NEEDS

*F1.* How would you rate your level of knowledge about the use of predictive genetic tests (features, eligibility criteria for testing, benefits, risks and non-medical implications)?

Not sufficient❒ Sufficient❒ Good❒ Excellent❒

*F2.* Do you think it is important to improve your knowledge on the use of predictive genetic tests in clinical and public health practice?

NO ❒ YES ❒

*F3.* Do you think that the teaching of the use of predictive genetic tests should be increased within the undergraduate course that you attended?

NO ❒ YES ❒

*F4.* Do you think that the teaching of the use of predictive genetic tests should be increased within the postgraduate School in Hygiene and Preventive Medicine?

NO ❒ YES ❒

*F5.* Do you think that specific postgraduate courses in predictive genetic testing for chronic diseases should be implemented?

NO ❒ YES ❒

**The questionnaire is finished. However, if you believe that there is something to add, please use the space below**

_________________________________________________________________________________________________________________________________________________________________________________________________________________________________________________________________________________________________________________________________________________________________________________________________________________________________________

_____________________________________________________________________________________
